# Fiberoptic Intubation Through a Laryngeal Mask in a Neonate With Difficult Airway: A Case Report

**DOI:** 10.7759/cureus.110992

**Published:** 2026-06-16

**Authors:** Rui Macedo-Campos, Marta Mugeiro, André Nogueira, Carla Pinto, Marta Coelho

**Affiliations:** 1 Anesthesiology, Unidade Local de Saúde Santa Maria, Lisbon, PRT; 2 Anesthesiology, Unidade Local de Saúde Algarve, Faro, PRT

**Keywords:** difficult airway management, fiberoptic intubation, laryngeal mask airway, neonatal airway management, pediatric anesthesia

## Abstract

Securing the airway in preterm patients remains one of the most demanding aspects of anesthesia due to their unique anatomical and physiological features. These differences require a tailored approach to minimize morbidity and mortality. We present the case of a preterm baby with no predictors of a difficult airway, scheduled for a transit reconstruction surgery. On the first endeavor, an unanticipated difficult airway was encountered, leading to a cardiac arrest, and the surgery had to be postponed.

On the second occasion, a difficult airway strategy was preemptively planned, and a multidisciplinary team was involved. The patient was successfully intubated using fiberoptic bronchoscopy through a laryngeal mask at the second attempt. This case underscores the importance of careful airway assessment, anticipation of difficulties, and the application of state-of-the-art airway management techniques in neonatal and pediatric anesthesia.

## Introduction

Despite remarkable technological and scientific advances, pediatric airway management - particularly in neonates - remains one of the greatest challenges in anesthesiology. It requires a long learning curve, and clinical experience plays a pivotal role when facing critical situations [1,2].

Several anatomical and physiological differences make the neonatal and pediatric airway inherently challenging [2,3]. Anatomically, the relatively large tongue, small oral cavity, cephalad and anterior larynx, and narrow subglottic region increase the difficulty of airway instrumentation and tracheal intubation. Premature infants present further difficulties due to reduced pulmonary development and a markedly lower physiological reserve [4].

Neonates demonstrate increased sensitivity to airway manipulation and irritative stimuli, with immature airway protective reflexes predisposing them to breath holding, laryngospasm, central apnea, bradycardia, and rapid hypoxemia [5]. These physiological responses are even more pronounced in premature infants, who exhibit reduced functional residual capacity, diminished pulmonary reserve, exaggerated hypoxic ventilatory depression, and increased susceptibility to apneic episodes in the setting of anesthetic administration or hypoxemia [5].

Airway management in this population is also associated with considerable procedural risk. Multicenter pediatric data have demonstrated first-attempt neonatal intubation success rates below 50%, with severe oxygen desaturation occurring in up to 48% of neonatal intensive care unit intubations and severe adverse airway events reported in approximately 4-5% of cases, including laryngospasm and the need for cardiac compressions [6]. These findings highlight the importance of careful airway planning, minimization of repeated intubation attempts, and the immediate availability of rescue airway devices and experienced personnel.

International guidelines emphasize the potentially catastrophic consequences of a difficult neonatal airway, highlighting the need for anticipatory planning, staff training, and immediate availability of appropriately sized airway devices and rescue supraglottic airways [7]. However, the evidence guiding neonatal airway management remains limited, given the rarity and emergent nature of these events [7].

## Case presentation

We describe the clinical course of a preterm male born at 27 weeks and 4 days of gestation due to severe preeclampsia. He was born with an extremely low birth weight (922 g) with no indications suggestive of malformation in the prenatal work-up. Immediately after delivery, he required endotracheal intubation for resuscitation; the airway management at that time is not detailed, but was described as uneventful. He remained intubated and mechanically ventilated in the neonatal intensive care unit.

By the age of 37 days, corrected age (CA) of 32 weeks and 2 days, in the context of abdominal distention and bloody stools, due to intestinal pneumatosis, the patient underwent a diverting ileostomy. As he remained intubated, no new airway interventions were necessary, and subsequent postoperative ventilation and extubation were uneventful.

At the age of 2 months and 2 days, CA of 36 weeks and 4 days, the patient was scheduled for gastrointestinal tract reconstruction. He was clinically stable on room air and required no vasopressor support. Following induction of general anesthesia, the first attempt at tracheal intubation was performed using a videolaryngoscope with a size-0 Macintosh blade (Supporting Healthcare NV; Oude Meer, The Netherlands), but intubation was unsuccessful. Facemask ventilation was effective, maintaining peripheral oxygen saturation (SpO₂) >98%. A second attempt, performed by the senior anesthesiologist in charge, was also unsuccessful, and a Cormack-Lehane grade of 4 was reported. Facemask ventilation then became more difficult, with desaturation to a minimum of 91%. A third attempt with a conventional laryngoscope and a size-0 Miller blade (HEINE Optotechnik; Gilching, Germany) again failed, with persistent grade 4 view. Severe desaturation to 80% and bradycardia ensued, progressing rapidly to cardiac arrest in asystole. Advanced life support was initiated immediately. An i-gel® size 1.0 laryngeal mask airway (LMA; Intersurgical Ltd., Wokingham, UK) was inserted to re-establish ventilation, and weight-adjusted epinephrine was administered. Return of spontaneous circulation (ROSC) was achieved after a single cycle of resuscitation, and surgery was postponed.

At 4 months of age (approximately 1 month CA, weight of 2.9 kg), the patient was scheduled for transit reconstruction surgery. Given the history of a difficult airway, a multidisciplinary team was involved, including two senior anesthesiologists, a neonatologist, and a pneumologist with proficiency in pediatric fibroscopy. Multiple devices were available to manage the airway, including a neonatal videolaryngoscope with different-size blades, an age-appropriate laryngeal mask, different-size endotracheal tubes, and a neonatal fiberoptic bronchoscope. From the multidisciplinary discussion, the strategy was defined with the main goal to preserve ventilatory drive. The first attempt would be an intubation through the fiberscope, a second attempt would be an intubation with the fiberscope through the LMA, and if those were not successful, a surgical airway would be performed at another time and the surgery postponed. The size compatibility of the devices was tested before the start of the procedure. 

**Figure 1:**
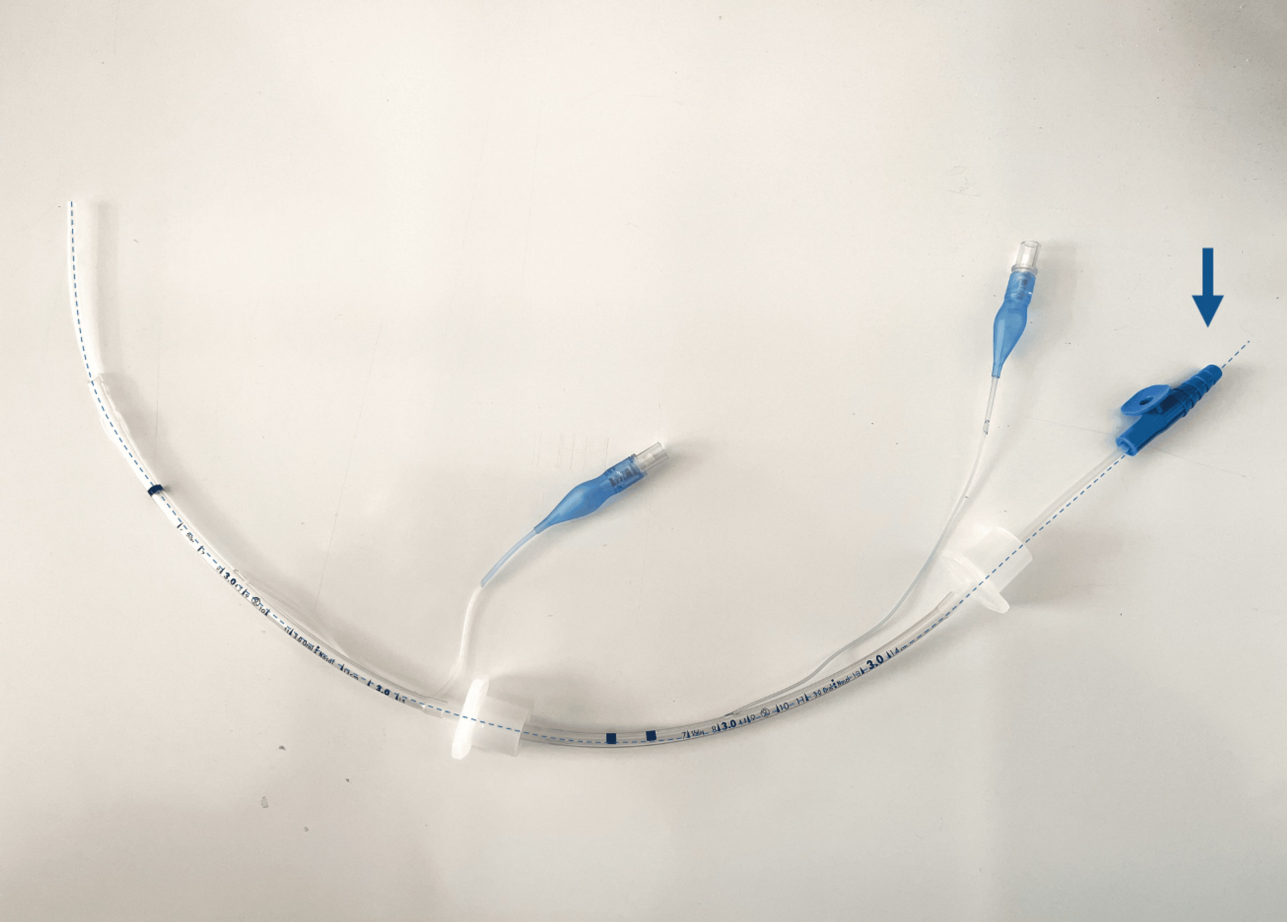
Assembly of Fiberoptic-Guided Intubation Using Two Endotracheal Tubes Schematic representation of the assembly using two consecutively mounted endotracheal tubes over the fiberoptic bronchoscope. The dashed line represents the internal channel of the fiberscope, and the blue arrow indicates its proximal end.

**Figure 2:**
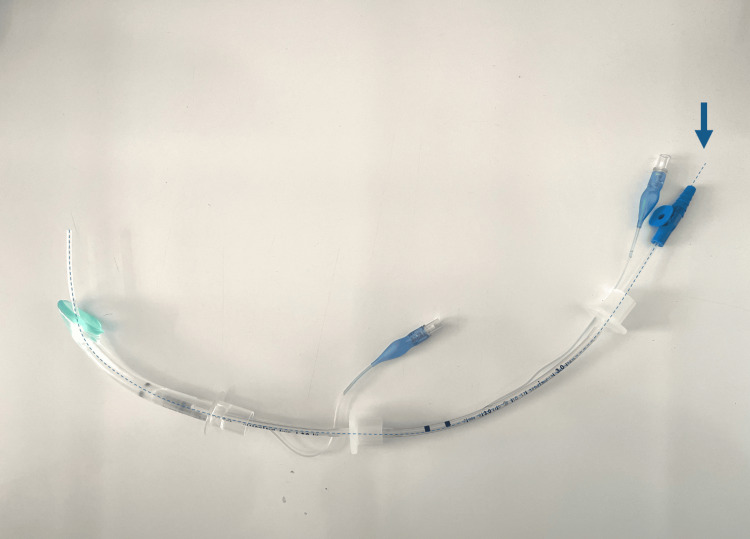
Fiberoptic-Guided Intubation Through a Laryngeal Mask Airway Schematic representation of the same assembly with the laryngeal mask airway in place. The dashed line represents the internal channel of the fiberscope, and the blue arrow indicates its proximal end.

Standard American Society of Anesthesiologists (ASA) monitoring was applied, and after inhalational induction, preserving ventilatory drive and with adequate analgesia, supplemental oxygen was continuously delivered via nasal cannulae throughout airway manipulation to optimize oxygenation. Following administration of intravenous lidocaine (2 mg) and fentanyl (3 µg), an attempt at endotracheal intubation was made with the bronchoscope, revealing epiglottic deformation and markedly anterior vocal cords. The advance of the fiberscope and endotracheal tube was unsuccessful due to sudden bronchospasm, resulting in desaturation and a new cardiac arrest. ROSC was attained after two minutes of effective cardiopulmonary resuscitation (CPR) and a single dose of epinephrine. Oxygenation was re-established using an LMA (i-gel® size 1.0). After stabilization, another intubation was attempted, this time with the fiberscope passing through the LMA, and a size 3.0 cuffed endotracheal tube was successfully placed. To slide the endotracheal tube through the LMA, two consecutive size 3.0 tubes were mounted on the fiberscope (as shown in Figures 1-2), and the upper one was used to slide the definitive one down with a Seldinger technique, using the fiberscope as the guide wire. The surgery proceeded under balanced general anesthesia, and the patient was transferred intubated to the pediatric intensive care unit. The patient remained intubated for 48 h after the procedure, and the extubation was uneventful. He was discharged to the ward four days following the surgery.

## Discussion

Pediatric airway management remains particularly challenging in neonates due to the combination of anatomical constraints and limited physiological reserve [2,7]. In premature infants, high oxygen consumption, reduced functional residual capacity, and increased susceptibility to hypoxemia predispose to rapid desaturation and cardiovascular collapse during airway manipulation [5,7]. In addition, repeated airway instrumentation may rapidly precipitate airway edema, worsening glottic visualization, and progressively increasing the difficulty of ventilation and intubation [7]. These mechanisms likely contributed to the deterioration observed in our patient after unsuccessful intubation attempts, ultimately culminating in severe hypoxemia and cardiac arrest. The second cardiac arrest was likely multifactorial, resulting from acute bronchospasm during fiberoptic manipulation, leading to abrupt deterioration in oxygenation. In this context, the severely reduced functional residual capacity and limited oxygen reserve of an extremely premature infant contributed to rapid desaturation and subsequent bradycardia progressing to cardiac arrest [5]. Furthermore, airway edema may have influenced the decision to maintain postoperative ventilation and delay extubation to ensure airway patency and patient safety in the immediate postoperative period.

In addition to these intrinsic factors, neonatal airway emergencies are frequently compounded by the limited availability of experienced personnel and appropriately sized airway equipment [7]. Furthermore, predictors of difficult airway validated in adults have limited applicability in neonates and infants, making unanticipated difficult-airway scenarios relatively common [1]. Consequently, repeated intubation attempts may rapidly worsen airway conditions through edema, bleeding, and progressive loss of effective ventilation, while significantly increasing the risk of severe hypoxemia and adverse cardiovascular events [7], with studies showing the number of intubation attempts as an independent factor associated with the occurrence of adverse events [8,9].

Current pediatric difficult airway guidelines therefore emphasize the importance of limiting repeated airway instrumentation, prioritizing oxygenation over tracheal intubation, and transitioning early to rescue strategies when difficulties arise [7]. Effective facemask ventilation and the use of supraglottic airway devices remain essential rescue measures in this population [7,9]. In our patient, insertion of an appropriately sized i-gel® laryngeal mask was critical for restoring oxygenation and allowed stabilization after cardiac arrest, highlighting the lifesaving role of supraglottic devices in neonatal airway emergencies.

Given the previous failed intubation attempts and the anticipated difficulty of direct laryngoscopy, a fiberoptic-guided intubation strategy was selected for the subsequent attempt. Maintaining spontaneous ventilation was considered essential to preserve oxygenation during airway manipulation and provide an additional safety margin in the event of prolonged or failed attempts. This approach aligns with current pediatric difficult airway recommendations, in which fiberoptic-assisted intubation is considered a valuable option when difficult airway management is anticipated, and preservation of ventilatory drive is desirable [5,7].

The use of a laryngeal mask airway as a conduit for fiberoptic intubation provided several advantages in this patient. Besides allowing continuous oxygenation and ventilation, the supraglottic device minimized repeated direct airway instrumentation and created a more stable path toward the glottic opening. This strategy was particularly relevant in the presence of distorted anatomy and in the context of a previously failed and critical airway with cardiorespiratory arrest episodes, in an extremely premature infant. Technical preparation was also crucial, including preprocedural compatibility testing between the bronchoscope, endotracheal tubes, and supraglottic device. This combined approach - fiberoptic bronchoscopy through a laryngeal mask airway following previous failed videolaryngoscopy and conventional laryngoscopy in a neonate with extreme prematurity and previous peri-arrest events - is rarely described and represents a noteworthy contribution to the limited literature on neonatal difficult airway management. Alternative supraglottic devices specifically designed as intubation conduits, such as the Air-Q®, may facilitate fiberoptic-guided intubation in neonates; however, their availability remains limited in many institutions.

This case also highlights the importance of multidisciplinary planning and human factors in neonatal airway management [1]. Following the first adverse event, the subsequent procedure involved a predefined airway strategy with participation of anesthesiologists, neonatologists, and a pediatric bronchoscopist, alongside preparation of multiple airway devices and predefined escalation plans. Such structured approaches are increasingly emphasized in pediatric airway management, as they improve team coordination, reduce cognitive overload during emergencies, and facilitate early transition between airway strategies [7,10].

Clear documentation of airway difficulties is equally essential to enable anticipation and adequate preparation for future interventions. In our patient, recognition and communication of the previous airway difficulty directly influenced the planning of the second procedure and likely contributed to its successful outcome. In addition, simulation-based airway training and regular multidisciplinary team drills have been associated with improved technical performance, communication, and reduction of adverse events in neonatal airway emergencies [10].

Finally, this case adds to the limited body of evidence regarding advanced airway management strategies in neonates. While both fiberoptic intubation and supraglottic airway devices are well described individually in pediatric airway management [7], their combined use as a planned rescue and definitive intubation strategy in extremely premature infants remains rarely reported. This case demonstrates that fiberoptic-guided intubation through a laryngeal mask airway can be an effective strategy in situations of anticipated and unanticipated difficult neonatal airway, particularly when conventional and videolaryngoscopic approaches have failed.

Importantly, this report also reinforces key principles in neonatal airway management, including the prioritization of oxygenation, the limitation of repeated airway attempts, and the value of early multidisciplinary planning. By sharing this experience, we aim to contribute to the growing evidence supporting structured, stepwise airway algorithms in this high-risk population and to encourage further refinement of specific guidelines for neonatal and pediatric difficult airway management.

## Conclusions

This case illustrates the inherent unpredictability and high-risk nature of neonatal airway management. Even in the absence of classic predictors, airway difficulty can arise abruptly and lead to critical complications. A structured multidisciplinary strategy involving anesthesiologists, neonatology support, and availability of pediatric airway expertise was fundamental in this case. Predefined airway plans, clear role allocation, and preparation of alternative airway devices allowed a controlled and stepwise approach during the second procedure. The outcome highlights the importance of incorporating lessons learned from the initial failed airway management, particularly in terms of anticipating difficulty and ensuring immediate access to rescue techniques.

Furthermore, this report contributes to the limited literature on neonatal difficult airway management, particularly regarding the use of fiberoptic intubation through a laryngeal mask as a safe and effective technique. Ongoing training, clear documentation, and shared experience among teams remain fundamental to improving outcomes in this vulnerable population.
